# Brain antioxidant markers, cognitive performance and acetylcholinesterase activity of rats: efficiency of Sonchus asper

**DOI:** 10.1186/1744-9081-8-21

**Published:** 2012-05-16

**Authors:** Rahmat Ali Khan, Muhammad Rashid Khan, Sumaira Sahreen

**Affiliations:** 1 Department of Biotechnology, Faculty of Biological Sciences, University of Science and Technology Bannu, Bannu, Pakistan; 2 Department of Biochemistry, Faculty of Biological Sciences, Quaid-i-Azam University Islamabad, Islamabad, Pakistan

**Keywords:** *Sonchus asper*, Cognitive performance, Acetylcholinesterase activity, Antioxidant enzymes

## Abstract

**Background:**

*Sonchus asper* (SA) is traditionally used as a folk medicine to treat mental disorders in Pakistan. The aim of this study was to investigate the effect of polyphenolic rich methanolic fraction of SA on cognitive performance, brain antioxidant activities and acetylcholinesterase activity in male rats.

**Methods:**

30 male Sprague–Dawley rats were equally divided into three groups in this study. Animals of group I (control) received saline (vehicle), group II received SA (50 mg/kg) body weight (b.w.), and group III treated with SA (100 mg/kg b.w.,) orally in dimethyl sulphoxide (DMSO) for 7 days. The effect of SA was checked on rat cognitive performance, brain antioxidatant and acetylcholinesterase activities. Evaluation of learning and memory was assessed by a step-through a passive avoidance test on day 6 after two habituation trials and an initial acquisition trial on day 5. Antioxidant potential was determined by measuring activities of superoxide dismutase (SOD), catalase (CAT), contents of thiobarbituric acid reactive substances (TBARS) and reduced glutathione (GSH) in whole-brain homogenates. Acetylcholinesterase (AChE) activity was determined by the colorimetric method.

**Results:**

Results showed that 100 mg/kg b.w., SA treated rats exhibited a significant improvement in learning and memory (step-through latency time). SA administration reduced lipid peroxidation products and elevated glutathione levels in the SA100-treated group. Furthermore, salt and detergent soluble AChE activity was significantly decreased in both SA-treated groups. Short-term orally supplementation of SA showed significant cognitive enhancement as well as elevated brain antioxidant enzymes and inhibited AChE activity.

**Conclusion:**

These findings stress the critical impact of *Sonchus asper* bioactive components on brain function.

## Background

Alzheimer’s disease (AD) is a slowly progressive disease of the brain that is characterized by the impairment of memory and eventually by disturbances in reasoning, planning, language, and perception. Amyloid β-peptide (Aβ) has been identified as a possible source of oxidative stress in AD because it can acquire a free-radical state that contributes to its toxic effects. Aβ-induced cytotoxicity is caused by intracellular accumulation of reactive oxygen species, which leads to lipid peroxidation and cell death [1]. Although the precise mechanisms by which Aβ induces neurotoxicity is still unknown, modulation of Aβ insult has been speculated to be an important preventive and neuro protective approach to control the onset of AD [2]. Use of antioxidants has been recognized as an effective method in minimizing pathological and toxic effects associated with Aβ-induced oxidative stress. Medicinal plants play a crucial role in the treatment of AD. *Ginkgo biloba* L. [3], *Huperzia serrata* (Thunb. Ex Murray) Trevis. [4] and salvianolic acid B [5] has been extensively investigated as natural therapeutic agents for the treatment of AD patients. Previous results revealed that memory dysfunction and cognitive deficits were significantly controlled with nutrient supplementation [6,7]. Supplementation of *Sonchus asper* revealed inhibition of inflammatory mediator and nitric oxide (NO) as well as lipid peroxidation in rats [8]. The central cholinergic system is essential for the regulation of cognitive functions, as evidenced by the extensive loss of cholinergic neurons observed in the forebrain of Alzheimer’s patients. Many anticholinergic drugs such as scopolamine cause learning and memory deficits in a variety of cognitive animal models [9,10]. Agonists of cholinergic receptors and inhibitors of AChE have been extensively used in order to increase endogenous acetylcholine levels and thus overcome cognitive deficits. Acetylcholinesterase metabolizes acetylcholine to choline and acetyl-CoA, which exist into asymmetric forms and globular (G) forms. The G1 form is cytosolic and G4 form is membrane bound by hydrophobic amino acid sequences or glycophospholipids. The detergent soluble (DS) and salt soluble (SS) fraction of AChE contains predominantly G4 and G1 forms [11].

Fruits, vegetables and medicinal plant bioactive metabolites play a key role in the slowing of many pathogenesis and neurodegenerative disorders such as Alzheimer and dementia [12,13]. Daily consumption of fresh vegetable reported in delaying of cognitive decline in older age [14]. Beneficial effects of these metabolites are proposed to associate with potentiating of antioxidant defenses, which is linked to normal aging and neurodegenerative diseases [15]. *Sonchus asper* (SA) has been traditionally used in the treatment of wound [12], oxidative dysfunction, bronchitis and asthma [13], central nervous system dysfunction, mental disorders [11], might be due to the presence of phenolic compounds, ascorbic acid and carotenoids [14,15]. Phenolic compounds have anticholinesterase activities, which might be valuable for the treatment of AD [16]. Due to the presence of phenolic compounds in SA, the present study was arranged to investigate the effect of SA on cognitive performance, antioxidant activities of brain homogenate, TBARS, GSH content and AChE activity.

## Materials and methods

### Plant material

Aerial parts (leaves, stem, flowers and seeds) of SA were collected and their specimen was submitted at Herbarium of Pakistan (Quaid-i-Azam University Islamabad, Pakistan). They were shade dried at room temperature, chopped and ground mechanically to a mesh size of 1 mm.

### Preparation of plant extracts

One kg dried sample of SA was extracted twice with 4 l of absolute methanol at 25°C. The extracts were filtrated through Whatman No. 1 fiter paper and concentrated using a rotary evaporator (Panchun Scientific Co., Kaohsiung, Taiwan) under reduced pressure at 40°C. The dried extract was stored at 4°C for in-vivo investigations.

### *In-vitro* acetylcholinesterase inhibition assay

The assay for AChE activity was conducted using the method of Ellman et al. [17], having acetylthiocholine iodide (ATCI) as a substrate. The rate of production of thiocholine is determined by the continuous reaction of the thiol with 5,5-dithiobis-2-nitrobenzoate (DTNB) ion to produce the yellow anion of 5-thio-2- nitro-benzoic acid. Briefly, in the 96well plates, 25 μl of 15 mM ATCI, 75 μl of 3 mM DTNB and 50 μl of 50 mM Tris HCl, pH 8.0, containing 0.1% bovine serum albumin (BSA), and 25 μl of SA (5–150 μg/ml) was added and the absorbance was measured at 405 nm after 5 min of incubation at room temperature. After 25 μl of 0.22 U/ml of AChE from electric eel (Sigma–Aldrich Corporation, St. Louis, MO, USA) was added, the absorbance was measured again after 5 min of incubation at room temperature. Percentage of inhibition was calculated by comparing the rate of enzymatic hydrolysis of ATCI for the samples to that of the blank (50% aqueous methanol in the buffer). Galanthamine (1–32 μM) was used as a reference standard and was supplied by Sigma–Aldrich. All determinations were carried out at least five times, and in triplicate, at each concentration of the standard and samples.

### Animals

30 Male, albino rats (180–190 g, b.w.), were provided by National Institute of Health Islamabad and were kept in ordinary cages at room temperature of 25 ± 3°C with a 12 h dark/light cycle. They were allowed free access to food in form of dry pellets and water and randomly divided into three groups: a vehicle group (*n* = 10) (Control), SA-treated group (*n* = 10) with SA 50 mg/kg b.w. (SA 50) and SA-treated group (*n* = 10) with 100 mg SA/kg b.w., (SA100). The SA extract was administered orally daily to SA50 and SA100 groups at a final volume 200 μl, while rats in the C group received 200 μl of saline. The study protocol was approved by Ethical Committee of Quaid-I-Azam University Islamabad for laboratory animal feed and care.

### 2.5. Behavioral testing: step-through a passive avoidance task

Rats were subjected to a step-through test on day 6, after a double training and an initial acquisition trial on day 5. It was performed according to previously described procedures, using a two-compartment passive avoidance apparatus (white/dark, separated by a black wall with a guillotine door in the middle part), with minor modifications of the time intervals. In detail, on day 5, the animals were allowed to habituate in the experimental room for 1 h prior to experiments. One hour later, each rat was placed in the illuminated chamber for the acquisition trial and was left to habituate to the apparatus. One hundred seconds later, the guillotine door was opened, and the animal was allowed to enter the dark compartment. The latency with which the animal crossed into the dark compartment was recorded. Animals that waited more than 100 s to enter the dark compartment were eliminated from the experiments. Once the animal crossed with all four paws to the next compartment, the guillotine door was closed, and the rat was taken into its home cage. The trial was repeated after 30 min as in the acquisition trial, where after 5 s the guillotine door was opened and as soon as the animal crossed to the dark compartment the door was closed and a foot shock (25 V, 3 mA, 5 s) was immediately delivered to the grid floor of the dark room. Thereafter, the rat was immediately removed from the apparatus and returned to its home cage. In this trial, the initial latency (IL) of entrance into the dark chamber was recorded (maximum time allowed was 120 s). Twenty-four hours after training, a retention test was performed to determine long-term memory. Each animal was placed in the light compartment for 20 s. The door was opened, and the step-through latency (STL) was measured for entering into the dark compartment. The test session ended when the animal entered the dark compartment or remained in the light compartment for 300 s (criterion for retrieval). During these sessions, no electric shock was applied. All training and testing sessions were carried out during the light phase between 08:00 and 14:00 h.

### Ex vivo assessment of antioxidant enzymes

After completions of experiment rats were killed, and whole intact brain was carefully removed and incubated on an ice chilled for cleaning. The cerebellum was separated immediately while rest of brain tissue was homogenized in a phosphate buffer (pH 7.6), centrifuged at 20,000 *× g,* 4◦C for 2 h to obtain a soluble salt part (SS). Re-extraction of the pellets was carried out to get a soluble detergent part (DS) [18]. The supernatant was collected and stored at −20◦C. Protein concentrations were determined by the Bradford assay with Bovine serum albumin as standard (0.05–1.00 mg/ml).

### AChE assessment

AChE activity was determined using the colorimetric assay of Ellman et al. [17], as previously described. Briefly, in the 96 well plates, 25 μl of 15 mM ATCI, 75 μl of 3 mM DTNB and 75 μl of 50 mM Tris–HCl, pH 8.0, containing 0.1% BSA, were added and the absorbance was read at 405 nm after five min incubation at room temperature. Any increase in absorbance due to the spontaneous hydrolysis of the substrate was corrected by subtracting the rate of the reaction before adding the enzyme. Then, 25 μl of sample (SS and DS fraction of brain homogenates) was added, and the absorbance was read again after 5 min of incubation at room temperature. The AChE activity is expressed as mol/min/g of tissue protein. All determinations were carried out twice and in triplicate.

### Catalase assay (CAT)

CAT activities were determined with reaction solution contained: 2.5 ml of 50 mmol phosphate buffers (pH 5.0), 0.4 ml of 5.9 mmol H_2_O_2_ and 0.1 ml tissue homogenate. Changes in absorbance of the reaction solution at 240 nm were determined after one minute. One unit of catalase activity was defined as an absorbance change of 0.01 as units/min [19].

### Superoxide dismutase assay (SOD)

SOD activity was estimated by the method of Kakar et al. [20]. Reaction mixture of this method contained: 0.1 ml of phenazine methosulphate (186 μmol), 1.2 ml of sodium pyrophosphate buffer (0.052 mmol; pH 7.0), 0.3 ml of the supernatant after centrifugation (1500 × g for 10 min followed by 10,000 × g for 15 min) of homogenate was added to the reaction mixture. Enzyme reaction was initiated by adding 0.2 ml of NADH (780 μmol) and stopped after 1 min by adding 1 ml of glacial acetic acid. Amount of chromogen formed was measured by recording color intensity at 560 nm. Results are expressed in units/mg protein.

### Glutathione-S-transferase assay (GST)

The glutathione-S-transferase activity mixture consisted of 1.475 ml phosphate buffer (0.1 mol, pH 6.5), 0.2 ml reduced glutathione (1 mmol), 0.025 ml 1-Chloro-2,4-dinitrobenzene (CDNB) (1 mmol) and 0.3 ml of homogenate in a total volume of 2.0 ml. The changes in the absorbance were recorded at 340 nm and enzymes activity was calculated as nmol CDNB conjugate formed/min/mg protein using a molar extinction coefficient of 9.6 × 10^3^ M^-1^ cm^-1^[21].

### Glutathione reductase assay (GSR)

Glutathione reductase activity was determined by method of Carlberg and Mannervik [22]. The reaction mixture consisted of 1.65 ml phosphate buffer: (0.1 mol; pH 7.6), 0.1 ml ethylenediaminetetraacetic acid (EDTA) (0.5 mmol), 0.05 ml oxidized glutathione (1 mmol), 0.1 ml nicotinamide adenine dinucleotide phosphate (NADPH) (0.1 mmol) and 0.1 ml of homogenate in a total volume of 2 ml. Enzyme activity were quantitated at 25°C by measuring disappearance of NADPH at 340 nm and was calculated as nmol NADPH oxidized/min/mg protein using a molar extinction coefficient of 6.22 × 10^3^ M^-1^ cm^-1^.

### Glutathione peroxidase assay (GSH-Px)

Glutathione peroxidase activity was assayed by the method of Mohandas et al. [23] (1984). The reaction mixture consisted of 1.49 ml phosphate buffer (0.1 mol; pH 7.4), 0.1 ml EDTA (1 mmol), 0.1 ml sodium azide (1 mmol), 0.05 ml glutathione reductase (1 IU/ml), 0.05 ml GSH (1 mmol), 0.1 ml NADPH (0.2 mmol), 0.01 ml H_2_O_2_ (0.25 mmol) and 0.1 ml of homogenate in a total volume of 2 ml. The disappearance of NADPH at 340 nm was recorded at 25°C. Enzyme activity was calculated as nmol NADPH oxidized/min/mg protein using a molar extinction coefficient of 6.22 × 10^3^ M^-1^ cm^-1^.

### Reduced glutathione assay (GSH)

Reduced glutathione was estimated by the method of Jollow et al. [24], using DTNB as a substrate. The yellow color developed was read immediately at 412 nm and expressed as μmol GSH/g tissue.

### Estimation of a lipid peroxidation assay (TBARS)

The assay for lipid peroxidation was carried out following the modified method of Iqbal et al. [25]. The reaction mixture in a total volume of 1.0 ml contained 0.58 ml phosphate buffer (0.1 mol; pH 7.4), 0.2 ml homogenate sample, 0.2 ml ascorbic acid (100 mmol), and 0.02 ml ferric chloride (100 mmol). The reaction mixture was incubated at 37°C in a shaking water bath for 1 h. The reaction was stopped by addition of 1.0 ml 10% trichloroacetic acid. Following addition of 1.0 ml 0.67% thiobarbituric acid, all the tubes were placed in boiling-water bath for 20 min and then shifted to crushed ice-bath before centrifuging at 2500 × g for 10 min. The amount of TBARS formed in each of the samples was assessed by measuring optical density of the supernatant at 535 nm using a spectrophotometer against a reagent blank. The results were expressed as nmol TBARS/min/mg tissue at 37°C using a molar extinction coefficient of 1.56 × 10^5^ M^-1^ cm^-1^.

### Statistical analysis

Data were expressed as standard error mean (Mean ± SEM) and one-way analysis of variance (ANOVA). The least significant difference (LSD) was determined using post hoc testing for inter group comparisons at a probability level of 0.05% and 0.01%. SPSS ver 14.0 (Chicago, IL, USA) and Microsoft Excel 2003 (Roselle, IL, USA) were used for the statistical and graphical evaluations.

## Results

### In vitro analysis of SA on AChE activity

**Figure 1:**
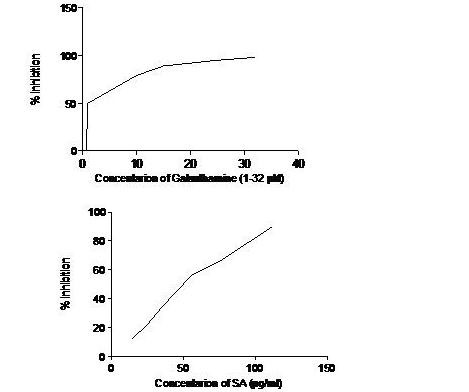
In vitro AChE inhibitory activity of SA (5–150 μg/ml) extract and Galanthamine (1–32 μM).

In order to check the direct inhibition of AChE, SA and galanthamine are used. Acetylcholinesterase degrades the neurotransmitter acetylcholine, producing choline and an acetate group. Figure 1 presents that SA exhibited moderate AChE inhibitory activity with an IC50 65 μg/ml while galanthamine an alkaloid extracted from *Galanthus nivalis* with an IC50 1.17 μM.

### Effect of SA extracts on the behavioral study

The changes of SA extract injected orally to rats for six days on the step-through learning capability is shown in Table 1. These changes are shown as the mean(s) of IL and STL for rats of each group. The STL involves relative resistant stimuli and is a direct measure of passive avoidance behavior. In the control, SA50 and SA100 groups, the IL was not significantly different (28 ± 3, 24 ± 3 and 28 ± 10 s), indicating that all groups behaved the same in the training trial. However, the SA100 group exhibited a significant increase (*p* > 0.05) in STL to 218 ± 50 s, 1 day after the acquisition trial, as compared to the control group (101 ± 32 s). No significant difference was observed in the SA50 group in comparison to the control group. Apparently, the SA extracts of 100 mg/kg b.w. facilitated learning in comparison to the control group, as is evident by the delay of transfer in the dark chamber.

**Table 1:** Showing IL and STL

**Treatment**	**IL**	**STL**
Control	28 ± 3	101 ± 32
SA50	24 ± 3	108 ± 50
SA100	28 ± 7	218 ± 50*

The effect of orally administration of 50 and 100 mg SA extract/kg BW on initial latency (IL) and step-through latency (STL) in the double trial step-through test. Asterisk (*) indicates significant difference from the control littermates (*n* = 10/group)

### Animal body and brain weight

No significant changes were observed in body weight of treated and control rats before, and after the administration of the SA extracts for seven days. Similarly, no considerable differences in the wet weight of the whole brain between control and SA treated rats (Table 2).

**Table 2:** Showing body weight before and after treatment and wet brain weight

**Treatment**	**Control**	**SA50**	**SA100**
Body weight before treatment (g)	186.2 ± 3.4	183.6 ± 4.2	188.2 ± 7.9
Body weight after treatment (g)	200.0 ± 5.4	189.5 ± 3.8	195.8 ± 7.2
Wet brain weight (mg)	362.2 ± 3.5	361.5 ± 5.1	357.1 ± 3.4

Mean ± S.E.M values (*n* = 10/group)

### The ex vivo effect of SA extracts on brain AChE activity

In order to determine AChE levels in the brain of rats, the soluble salt part (SS) and detergent soluble part (DS) fractions of brain homogenates were assayed using the colorimetric method of Ellman et al. [17] (Table 3). Short-term SA administration (SA50 and SA100) resulted in a significant decrease in AChE specific activity in both SS and DS fractions as compared to control.

**Table 3:** **Effect of orally *Sonchus asper *(SA) administration for 7 days on ex vivo acetylcholinesterase (AChE) activity (mol/min/g of tissue protein) in rat brain**

**Treatment**	**Salt soluble (SS)-AChE**	**Detergent soluble (DS)-AChE**
Control	0.175 ± 0.0057	0.889 ± 0.074
SA50	0.101 ± 0.002*	0.525 ± 0.031**
SA100	0.074 ± 0.010**	0.300 ± 0.051**

* *p* < 0.05 significant difference from control

** *p* < 0.01 significant difference from control

The AChE activity for each group denotes mean ± S.E.M values

### Effect of SA extracts on brain oxidative status

Table 4 shows changes in brain activities of antioxidant enzymes, glutathione and TBARS contents in all the experimental groups of rat. Administration of SA significantly (*p* < 0.05) altered the concentration of SOD, CAT and TBARS in a dose-dependent way. In particular, SA administration at 100 mg/kg b.w. almost reduced the concentration of TBARS in brain tissue. Additionally, the levels of GSH and activities of GST, GSR and GSHpx were significantly (*p* < 0.05) increased in the brain tissue of SA50 and SA100-treated rats.

**Table 4:** Effect of orally SA administration for 7 days in biochemical parameters of rat brain antioxidant status

**Treatment**	**Catalase (U/min)**	**Superoxide dismutase (U/mg protein)**	**Glutathione peroxidase assay (nmol/mg protein)**	**Glutathione reductase (nmol/min** **/mg protein)**	**Glutathione-S-transferase (nmol/min** **/mg protein)**	**Reduced glutathione (μmol/g tissue)**	**Thiobarbituric acid reactive substances (nmol/min** **/ mg protein)**
Control	11.0 ± 0.25	7.18 ± 2.8	43.3 ± 3.58	174.5 ± 20.7	115.8 ± 26.11	64.5 ± 10.7	184.5 ± 8.7
SA50	15.5 ± 2.8*	13.5 ± 1.4**	55.3 ± 1.85*	203.3 ± 10.3**	139.8 ± 13.3 *	88.0 ± 8.3*	200.3 ± 7.3*
SA100	14.0 ± 1.12*	18.3 ± 1.7 **	65.3 ± 5.14**	218.0 ± 10.5 **	150.8 ± 11.5**	91.8 ± 10.0**	208.0 ± 10.0**

* *p* < 0.05 significant difference from control littermates (*n* = 10/group)

** *p* < 0.01 significant difference from control littermates (*n* = 10/group)

The rats brain biochemical parameters are expressed as mean ± S.E.M values

## Discussion

The present study has been carried out using rats for investigation of learning task. The passive avoidance task is a fear-aggravated test used to evaluate learning and memory in rodent models of CNS disorders. In this test, subjects learn to avoid an environment in which an aversive stimulus (such as a foot-shock) was delivered. On 5^th^ day the IL to enter the dark chamber is measured as a control for visual ability and motor activity. In our experiment, the mean IL values did not differ among the different groups. The STL is a measure of the memory of the aversive experience. The mean STL of rat treated with the high concentration of the SA extract (SA100) was significantly higher than those of the other groups on day 6. These results are in agreement with recent reports on the long-term memory enhancing effect in the inhibition avoidance test of dietary supplementation with *Vaccinium ashei* (Ericaceae) (rabbiteye blueberries) of 3-month-old mice for 30 days [26]. Data of the present study revealed that *Sonchus asper* significantly decreased brain AChE activity in rats. This result supports the ideas, which might be due to a decrease in gene transcription, translation and enhance cholinergic activity thereby improving cognitive function [27]. AChE activity was decreased in both SS and DS fractions, with significant effects in rats treated with SA 100 comparatively SA50 rats treated group. Oxidative stress and antioxidant system play an important role in pathophysiological cerebral changes. Evaluation of the protective effect *Sonchus asper* on rat brain oxidative stress parameters and antioxidant mechanisms was an important aim of our study. The activity of SOD is a sensitive index in oxidative damage as it scavenges the superoxide anion to form hydrogen peroxide leading to diminish the toxic effects. The catalase is enzymatic antioxidants, widely distributed in all animal tissues that decompose hydrogen peroxide and protect the tissue from highly reactive hydroxyl radicals. Data revealed that administration of both SA50 and SA100 increased the activity of SOD and CAT as reported during supplementation *Sonchus asper* in rat [8].

Glutathione reduced, glutathione reductase and glutathione-S-transferase is thought to be the fundamental antioxidant enzymes, for they are closely related to the direct elimination of reactive oxygen species. Therefore, the reduction in the activity of these enzymes may result in a number of deleterious effects due to the accumulation of superoxide radicals and hydrogen peroxide, linked with neurodegenerative diseases [28]. Supplementation of SA100 and SA50 for seven days improved the activity, showing protection against free radicals. From these results, it was inferred that administration of SA in healthy rat attenuated brain oxidative damages, increased activity of antioxidant enzymes, GSH and AChE while decreased TBARS level. These antioxidant effects of SA were might be associated with the enhancement in performance in the passive avoidance behavioral test. Although the mechanisms underlying these effects are still unknown and require more pharmacological, neurochemical and pharmacokinetic research to establish any therapeutic advantage. However excessive use of SA is could be suggested for the prevention of cognitive decline during aging and neurodegenerative disease.

## Competing interests

The authors declare that they have no competing interests.

## Authors’ contributions

RAK made a significant contribution to acquisition of and analyses of data and drafting of the manuscript. MRK made a substantial contribution to the conception and design of the study, interpretation of data, as well as drafting and revising of the manuscript. SS participated in the study design as well as the collection and analyses of data. All authors read and approved the final manuscript.

## References

[B1] Kumar U Dunlop DM Richardson JS The acute neurotoxic effect of β-amyloid on mature cultures of rat hippocampal neurons is attenuated by the anti-oxidant U-78517 F Intl J neurosci 1994 1994 79 185 190 10.3109/00207459408986079 7744560

[B2] Kim DSHL Park SY Kim JK Curcuminoids from *Curcuma longa* L. (Zingiberaceae) that protect PC12 rat pheochromocytoma and normal human umbilical vein endothelial cells from βA(1–42) insult Neurosci Letters 2001 303 57 61 10.1016/S0304-3940(01)01677-9 11297823

[B3] Oken BS Storzbach DM Kaye JA The efficacy of *Ginkgo biloba* on cognitive function in Alzheimer disease Arch Neurol 1995 55 1409 14015 9823823 10.1001/archneur.55.11.1409

[B4] Skolnick AA Old Chinese herbal medicine used for fever yields possible new Alzheimer disease therapy J Am Med Assoc 1997 277 776 10.1001/jama.1997.03540340010004 9052690

[B5] Durairajan SS Yuan Q Xie L Chan WS Kum WF Koo I Liu C Song Y Huang JD Klein WL Li M Salvianolic acid B inhibits Aβ fibril formation and disaggregates preformed fibrils and protects against Aβ-induced cytotoxicty Neurochem Intl 2008 52 741 750 10.1016/j.neuint.2007.09.006 17964692

[B6] Joseph JA Shukitt-Hale B Denisova NA Bielinski D Martin A McEwen JJ Reversals of age-related declines in neuronal signal transduction, cognitive, and motor behavioural deficits with blueberry, spinach, or strawberry dietary supplementation J Neurosci 1999 19 8114 8121 10479711 10.1523/JNEUROSCI.19-18-08114.1999 PMC6782471

[B7] Shukitt-Hale B Lau FC Joseph JA Berry fruit supplementation and the aging brain J Agric Food Chem 2008 56 636 641 10.1021/jf072505f 18211020

[B8] Khan RA Khan MR Sahreen S Bokhari J Prevention of CCl4-induced nephrotoxicity with *Sonchus asper* in rat Food Chem Toxicol 2010 48 2469 2476 10.1016/j.fct.2010.06.016 20550952

[B9] Sarter M Bruno JP Cognitive functions of cortical acetylcholine: toward a unifying hypothesis Brain Res Rev 1997 23 28 46 10.1016/S0165-0173(96)00009-4 9063585

[B10] Zimmerman G Soreq H Termination and beyond: acetylcholinesterase as a modulator of synaptic transmission Cell Tissue Res 2006 326 655 669 10.1007/s00441-006-0239-8 16802134

[B11] Lane RM Potkin SG Enz A Targeting acetylcholinesterase and butyrylcholinesterase in dementia Intl J Neuropsycopharmacol 2006 9 101 124 10.1017/S1461145705005833 16083515

[B12] Rehman EU Indigenous knowledge on medicinal plants, village Barali Kass and its allied areas, District Kotli Azad Jammu and Kashmir, Pakistan Ethno Leaflets 2006 10 254 264

[B13] Dai Q Borenstein AR Wu Y Jackson JC Larson EB Fruit and vegetable juices and Alzheimer’s disease: the Kame Project Am J Med 2006 119 751 759 10.1016/j.amjmed.2006.03.045 16945610 PMC2266591

[B14] Letenneur L Proust-Lima C Le Gouge A Dartigues JF BPrBerger-Gateau P Flavonoid intake and cognitive decline over a 10-year period Am J Epidemiol 2007 165 1364 1371 10.1093/aje/kwm036 17369607

[B15] Morris MC Evans DA Tangney CC Bienias JL Wilson RS Associations of vegetable and fruit consumption with age-related cognitive change Neurology 2006 67 1370 1376 10.1212/01.wnl.0000240224.38978.d8 17060562 PMC3393520

[B16] Tumiatti V Bolognesi ML Minarini A Rosini M Milelli A Matera R Progress in acetylcholinesterase inhibitors for Alzheimer’s disease: an update Expert Opinion on Therapeutic Patents 2008 12 45 56 10.1517/14728222.12.1.45

[B17] Ellman GL Courtney KD Andres V Featherstone RM A new and rapid colorimetric determination of acetylcholinesterase activity Biochem Pharmacol 1961 7 88 95 10.1016/0006-2952(61)90145-9 13726518

[B18] Kameyama T Nabeshima T Kozawa T Step-down-type passive avoidance- and escape-learning method. Suitability for experimental amnesia models J Pharmacol Methods 1986 16 39 52 10.1016/0160-5402(86)90027-6 3747545

[B19] Chance B Maehly AC Assay of catalase and peroxidases Met Enzymol 1955 11 764 775

[B20] Kakkar P Das B Viswanathan PN A modified spectrophotometric assay of superoxide dismutase Indian J Biochem Biophys 1984 21 130 132 6490072

[B21] Habig WH Pabst MJ Jakoby WB Glutathione-S-transferases: the first enzymatic step in mercapturic acid formation J Biol Chem 1974 249 7130 7139 4436300

[B22] Carlberg I Mannervik EB Glutathione level in rat brain J Biol Chem 1975 250 4475 4480 237922

[B23] Mohandas J Marshal JJ Duggin GG Horvath JS Tiller DJ Differential distribution of glutathione and glutathione-related enzymes in rabbit kidney. Possible implications in analgesic nephropathy Biochem Pharmacol 1984 33 1801 1807 10.1016/0006-2952(84)90353-8 6145422

[B24] Jollow DJ Mitchell JR Zampaglione N Gillete JR Bromobenzene induced liver necrosis. Protective role of glutathione and evidence for 3, 4-bromobenzene oxide as a hepatotoxic metabolite Pharmacol 2003 11 151 169 10.1159/000136485 4831804

[B25] Iqbal M Sharma MD Zadeh HR Hasan N Abdulla M Athar M Glutathione metabolizing enzymes and oxidative stress in ferric nitrilotriacetate (Fe-NTA) mediated hepatic injury Redox Report 1996 2 385 391 10.1080/13510002.1996.11747079 27406673

[B26] Sabeen M Ahmad AA Exploring the Folk Medicinal Flora of Abbotabad City, Pakistan Ethno Leaflets 2009 13 810 833

[B27] Shahidi S Komaki A Mahmoodi M Atrvash N Ghodrati M Ascorbic acid supplementation could affect passive avoidance learning and memory in rat Brain Res Bull 2008 76 109 113 10.1016/j.brainresbull.2008.01.003 18395619

[B28] Fang YZ Yang S Wu G Free radicals, antioxidants, and nutrition Nutrition 2000 18 872 879 12361782 10.1016/s0899-9007(02)00916-4

